# The effect of oxygen tension on human articular chondrocyte matrix synthesis: Integration of experimental and computational approaches

**DOI:** 10.1002/bit.25241

**Published:** 2014-05-05

**Authors:** S Li, ROC Oreffo, BG Sengers, RS Tare

**Affiliations:** 1 Centre for Human Development, Stem Cells and Regeneration, Faculty of Medicine, University of SouthamptonSouthampton, Hampshire, UK; 2 Bioengineering Science, Faculty of Engineering and the Environment, University of Southampton, HighfieldSouthampton, SO17 1BJ, Hampshire, UK

**Keywords:** cartilage tissue engineering, human articular chondrocytes, pellet culture, oxygen tension, mathematical modeling, image analysis

## Abstract

Significant oxygen gradients occur within tissue engineered cartilaginous constructs. Although oxygen tension is an important limiting parameter in the development of new cartilage matrix, its precise role in matrix formation by chondrocytes remains controversial, primarily due to discrepancies in the experimental setup applied in different studies. In this study, the specific effects of oxygen tension on the synthesis of cartilaginous matrix by human articular chondrocytes were studied using a combined experimental-computational approach in a “scaffold-free” 3D pellet culture model. Key parameters including cellular oxygen uptake rate were determined experimentally and used in conjunction with a mathematical model to estimate oxygen tension profiles in 21-day cartilaginous pellets. A threshold oxygen tension (pO_2_ ≈ 8% atmospheric pressure) for human articular chondrocytes was estimated from these inferred oxygen profiles and histological analysis of pellet sections. Human articular chondrocytes that experienced oxygen tension below this threshold demonstrated enhanced proteoglycan deposition. Conversely, oxygen tension higher than the threshold favored collagen synthesis. This study has demonstrated a close relationship between oxygen tension and matrix synthesis by human articular chondrocytes in a “scaffold-free” 3D pellet culture model, providing valuable insight into the understanding and optimization of cartilage bioengineering approaches. Biotechnol. Bioeng. 2014;111: 1876–1885.

## Introduction

The limited capacity of adult articular cartilage to self-repair has led to considerable interest in the development of effective cell-based therapies and cartilage tissue engineering strategies to treat cartilage defects in an increasing aging population. The chondrogenic potential of articular chondrocytes is dependent on a complex array of environmental, biochemical, and physical factors. Chondrocytes grown in monolayer cultures gradually lose their differentiated chondrogenic phenotype and become fibroblastic, a process known as dedifferentiation, which is characterized by significant down-regulation of chondrocyte phenotypic markers (Benya and Shaffer, 1982; Lin et al., 2008). Three-dimensional (3D) culture environments are therefore routinely applied to induce and maintain the chondrogenic potential of isolated multipotent stem cells and chondrocytes. In addition to biomaterial scaffold-based approaches, “scaffold-free” tissue engineering techniques have been developed to generate cartilaginous constructs or chondrospheres by culturing chondrocytes in high-density 3D pellets/aggregates (Adkisson et al., 2001; Schubert et al., 2009; Tare et al., 2005).

Within tissue engineered cartilaginous explants, the exchange of nutrients (e.g., glucose and oxygen) and metabolic by-products (e.g., lactic acid) can only occur through diffusion unless active fluid perfusion is applied (Malda et al., 2004). The difference between diffusion and cellular metabolism will inevitably lead to the formation of gradients in metabolite concentrations that can significantly affect cellular behavior including cell viability, metabolism, and matrix synthesis (Lee and Urban, 1997; Zhou et al., 2008). Given the complexities and challenges of monitoring these gradients in situ, significant efforts have been devoted to the development of appropriate mathematical models to evaluate them (Sengers et al., 2007b). Oxygen gradients have been modeled either alone (Malda et al., 2004) or in combination with glucose and lactic acid (Sengers et al., 2005a; Zhou et al., 2008) for tissue-engineered cartilaginous explants. However, only a limited number of studies have reported the relationship between the model prediction and experimentally evaluated chondrogenesis.

This study utilized a multidisciplinary approach, which combined 3D “scaffold-free” pellet culture technique, mathematical modeling and image analysis, to determine the effects of oxygen tension on cartilaginous matrix synthesis by human articular chondrocytes (HACs).

## Materials and Methods

Chemicals and reagents were purchased from Invitrogen (Paisley, UK) or Sigma–Aldrich (Gillingham, UK) unless specified otherwise. Human femoral head samples were obtained from four haematologically normal Osteoarthritic individuals (three male and one female, mean age: 74 ± 14.88 years) following routine total hip replacement surgery. Only tissue that would have been discarded was used in this study, with the approval of the Southampton and South West Hampshire Research Ethics Committee (LREC 194/99/1 & 210/01).

### Chondrocyte Isolation and Cell Expansion

HACs were isolated by sequential enzymatic digestion of deep-zone articular cartilage pieces dissected from the non load-bearing region of the femoral heads (de Andres et al., 2011). In brief, cartilage pieces were sequentially digested using 500 µg/mL trypsin-EDTA for 30 min, 1 mg/mL hyaluronidase for 15 min and 10 mg/mL collagenase B (ROCHE Diagnostics, Burgess Hill, UK) overnight on a rotating mixer at 37°C. Isolated chondrocytes were cultured to confluence in monolayer cultures in α-MEM supplemented with 10% (v/v) FCS, 100 unit/mL penicillin, 100 µg/mL streptomycin, and 100 µM ascorbate 2-phosphate. Cultures were maintained in humidified atmosphere at 37°C, 5% CO_2_, and 21% O_2_.

### Pellet Culture

Pellet cultures were performed in accordance with the protocol published previously (Tare et al., 2005). Serum-free chondrogenic media was made up of α-MEM supplemented with 10 ng/mL rhTGF-β3 (PeproTech, London, UK), 100 µM ascorbate-2-phosphate, 10 nM dexamethasone and 1× ITS liquid supplement (10 µg/mL insulin, 5.5 µg/mL transferrin, and 5 ng/mL selenite premix), a modification of the media used previously (Mackay et al., 1998; Malpeli et al., 2004). Monolayer cultured HACs were harvested at confluence and suspended in serum-free chondrogenic media at concentrations of 0.6 × 10^4^, 1 × 10^5^, 2 × 10^5^, 5 × 10^5^, and 1 × 10^6^ cells/mL. One milliliter of cell suspension was added to each sterile 25 mL polycarbonate universal tube and centrifuged at 400G for 5 min at 4°C. The resulting cell pellet was not dispersed and cultured in humidified atmosphere at 37°C, 5% CO_2_, and 21% O_2_ for 21 days. Chondrogenic media changes were carried out every 2 days over the 21-day culture period. Pellets were fixed overnight in 4% paraformaldehyde (PFA) at the end of the 21-day culture period. For each patient (*n* = 4), two pellets were generated using each of the above mentioned cell concentrations.

### Processing, Embedding, and Section Cutting

PFA-fixed samples were processed through graded ethanol (50–100%) and histoclear (100%) prior to embedding in paraffin wax. Sequential sections (7 µm thick) were cut through approximately 25%, 50%, and 75% of the sample and mounted on glass microscope slides.

#### Alcian Blue and Sirius Red

Following nuclear staining with Weigert's haematoxylin, sections were stained with Alcian blue 8GX (5 mg/mL in 1% (v/v) glacial acetic acid) and Sirius red F3B (10 mg/mL in saturated picric acid). Sets of three sequential sections were stained with Alcian blue only, Sirius red only, and both stains. Stained samples were imaged with the Olympus dotSlide system (Olympus Microscopy, Southend-on-Sea, UK).

### Image Analysis

#### Pellet Radius and Volume

Images of sequential sections of each pellet were loaded into the image analysis software ImageJ (NIH, Bethesda, MD). The “Analyze Particle” tool in ImageJ was used to trace the complete outline of the cartilaginous pellet section and to determine the enclosed area (SA_sec_) of the section (Fig. S1). Assuming a completely spherical pellet, the radius of each individual pellet section (*r*_sec_) was calculated from a circle with the same SA_sec_ as the cartilaginous pellet section. The largest radius of all sequential sections was denoted as the radius of the pellet, *r*_max_. The pellet volume (*V*_total_) was then calculated from *r*_max_.

#### Pellet Cell Number and Cell Density

A custom programmed script in MatLab was used to identify and count the number of cell nuclei counter-stained with Weigert's haematoxylin in each section (Fig. S2a,b) (Sengers et al., 2007a). Sequential sections stained only with Sirius red were used due to the high contrast between the cell nuclei and background matrix staining. Pellet cell density (*ρ*_cell_) was calculated by dividing the total number of cells/cell nuclei in all Sirius red-stained 7 µm-thick sections of the pellet by the sum of the individual volumes of these sections. The same method was used to determine the local cell density in zonal regions with equal width of 50 µm (Fig. S2c). Given that the same nuclei can appear in more than one successive histological section, a correction was made to the “raw” cell density data as previously described (Abercrombie, 1946). In brief, *ν*_a_ = *ν*_c_ × *t*/(*d* + *t*), where *ν*_a_ was the corrected number of nuclei per section, *ν*_c_ was the raw count of number of nuclei per section, *t* was the thickness (in µm) of the section, and *d* the average diameter (in µm) of the nuclei.

#### Pellet Morphology Analysis—Measurement of the Thickness of Fibrous Collagenous Band Around the Pellet Periphery

Images of pellet sections stained with either Alcian blue or Sirius red alone were used to determine the thickness of the fibrous collagenous band around the periphery of the pellet. In brief, each individual color image consisting of the three RGB (red, green, blue) channels was converted into three gray-scale images (*I*_R_, *I*_G_, *I*_B_) (Fig. S3a,b). Visually red stained areas have high values of *I*_R_. To enhance color specificity, that is, to extract the desired red areas and eliminate any white or gray areas, the *I*_G_ channel was subtracted from the *I*_R_ channel, resulting in the staining intensity measure *I*_RS_ (*I*_RS_ = *I*_R_ − *I*_G_). Pixels with negative values were converted to 0, that is, black pixels. The resulting image therefore contained only the gray-scale pixels (value = 0–255) representing the intensity of the red staining (Fig. S3c top image), wherein intensely stained areas were represented by white pixels, lighter areas were represented by gray pixels and the remainder of the image were represented by black pixels. The reconstructed Sirius red images were loaded into ImageJ for the measurement of the thickness of the fibrous collagenous band (Fig. S4). A similar procedure was followed to extract the desired blue stained areas, using *I*_BS_ = *I*_B_ − *I*_R_ (Fig. S3c bottom image). Furthermore, color intensity profiles were also plotted for both reconstructed Alcian blue and Sirius red images.

### Measurement of Cellular Oxygen Uptake Rate

HAC oxygen uptake rate (*Q*_cell_) was measured using the BD OBS oxygen biosensor system (BD Biosciences, Oxford, UK) as previously described (Forristal et al., 2013; Guarino et al., 2004). For each patient sample, three replicates were performed for the measurement of *Q*_cell_. HACs were seeded into the wells of an OBS microplate at a density of 500,000 cells/well in chondrogenic media. The wells were sealed with airtight aluminium film to prevent gas exchange. The OBS microplate was read on a FLUOstar OPTIMA fluorescence microplate reader (BMG Labtech, Aylesbury, UK) at 485 nm excitation and 620 nm emission wavelengths. The OBS microplate was maintained at 37°C inside the fluorescence microplate reader. The intensity of emitted fluorescence varied inversely with oxygen concentration and was recorded every 2 min during the 120 min measurement period. Plain α-MEM, which was equilibrated with air at 37°C, was used as “no-cell” standard oxygen concentration control (*I*_a_, ambient fluorescence intensity). α-MEM with 200 mM sodium sulfite was used as 0% oxygen control (*I*_0_, maximum fluorescence intensity). The 0% oxygen control was prepared 30 min prior to each test to ensure all oxygen was removed by sodium sulfite.

#### Data Normalization and Calculation of Oxygen Concentration and Consumption Rate

As the fluorescence intensity measurements can vary with the concentration of the fluorophore and machine drift, a two-step normalization was performed (Guarino et al., 2004). The raw fluorescence unit (RFU) of each well was normalized to the value of ambient controls (*I*_a_), yielding the so-called NRF (normalized relative fluorescence) value (NRF = RFU/*I*_a_). The dynamic range (DR = *I*_0_/*I*_a_), a constant for the given experiment setup (e.g., temperature, wavelength), accounted for any fluctuation in intensity between the time points due to machine drift. This double normalization yielded a drift- and concentration-corrected fluorescence intensity, which was then used to calculate the equilibrium oxygen concentration at the bottom of each well at a given time point, where [O_2_] = (DR/NRF − 1)/Ksv. Ksv, the Stern–Volmer constant, was a function for quenching of the fluorophore by oxygen (Ksv = (DR − 1)/[O_2_]_a_). [O_2_]_a_ was the ambient oxygen concentration in the medium at equilibrium, which was 195 µM at 37°C in 5% CO_2_ environment (Guarino et al., 2004; Wang et al., 2005). The graph of oxygen tension versus time during the 120-min measurement period (Fig. S5) was used to determine the cellular oxygen uptake rate, *Q*_cell_ = ([O_2_]_T2_ − [O_2_]_T1_)/(T_2_ − T_1_)/(number of cells in well), which represented the amount of oxygen consumed per cell in a given time period and was expressed in mol s^−1^ cell^−1^.

### Statistical Analysis

All results were presented as mean ± SD. Statistical analysis was performed using Mann–Whitney *U*-test with Bonferroni correction. Results were deemed significant if the probability of occurrence by random chance alone was less than 5% (i.e., *P* < 0.05).

### Mathematical Model to Predict Oxygen Tension Profile

In the present study, the equilibrium between oxygen diffusion and consumption was represented as a diffusion–reaction equation. The oxygen tension *C*(*r*) [mol cm^−3^] in spherical coordinates was governed by the solute mass balance including Fick's law of diffusion, assuming (i) spherical symmetry, (ii) Michaelis–Menten kinetics (Hiltmann and Lory, 1983), and (iii) steady-state:
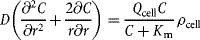
1subject to boundary conditions: (∂*C*/∂*r*) = 0 for *r* = 0 (2a) and *C* = *C*_0_ for *r* = *r*_max_ (2b).

Here, *r* [cm] was the distance from the centre of cartilaginous pellet, *D* [cm^2^ s^−1^] was the effective diffusion coefficient of oxygen, *Q*_cell_ [mol cell^−1^ s^−1^] was the maximum cellular oxygen uptake rate, *K*_m_ [mol cm^−3^] was the Michaelis–Menten constant, *ρ*_cell_ [cell cm^−3^] was the cell density, *C*_0_ [mol cm^−3^] was the ambient oxygen concentration (constant) on the surface of the cartilaginous pellet, *r*_max_ [cm] was the radius of cartilaginous pellet.

Equation (1) was solved numerically, utilizing experimentally determined values for *ρ*_cell_, *r*_max_, and *Q*_cell_, to predict the oxygen tension profiles from the periphery to the centre of cartilaginous pellets. A custom finite element method script was used in MATLAB (Sengers et al., 2007a).

## Results

### Measurement of Cartilaginous Pellet Volume

**Figure 1:**
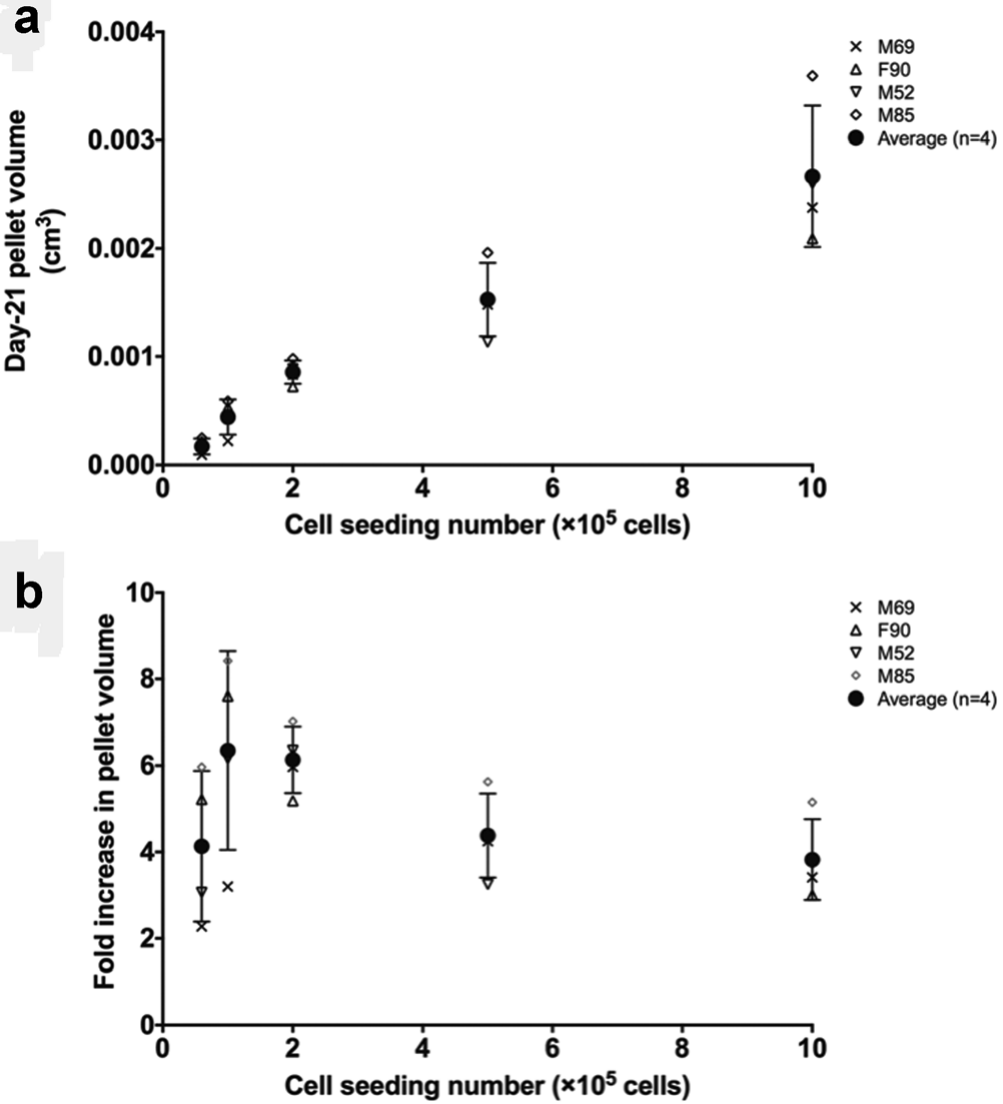
The relation between initial cell seeding number and day-21 HAC cartilaginous pellet volume. Higher initial cell seeding number led to larger total pellet volume (a). However, the largest volume increase, when compared to initial cell aggregate volume, was observed at cell seeding numbers of 1 × 10^5^ and 2 × 10^5^ (b).

Higher initial cell seeding numbers resulted in larger total volumes for cartilaginous pellets harvested after the 21-day culture period (Fig. 1a, pellet radii (*r*_max_) listed in Table S1). However, when the total volumes of the Day-21 cartilaginous pellets were compared to the initial (i.e., Day-1) volumes of the cell aggregates, the largest fold increase in volume (≈6-fold) was observed when initial cell seeding numbers of 1 × 10^5^ and 2 × 10^5^ were used for pellet generation (Fig. 1b) (Fold volume increase = Day-21 pellet volume/Day-1 pellet volume). The pellet volume on day-1 was considered as the volume of the cell aggregate without any matrix (i.e., Day-1 pellet volume = volume of individual chondrocyte × seeding cell/chondrocyte number used to generate the pellet). The average chondrocyte radius of 5.49 ± 0.51 µm was measured directly using ImageJ. This value was comparable to the average size of chondrocytes (from healthy joints) reported in published literature (Leipzig and Athanasiou, 2008; Stockwell, 1971).

**Figure 2:**
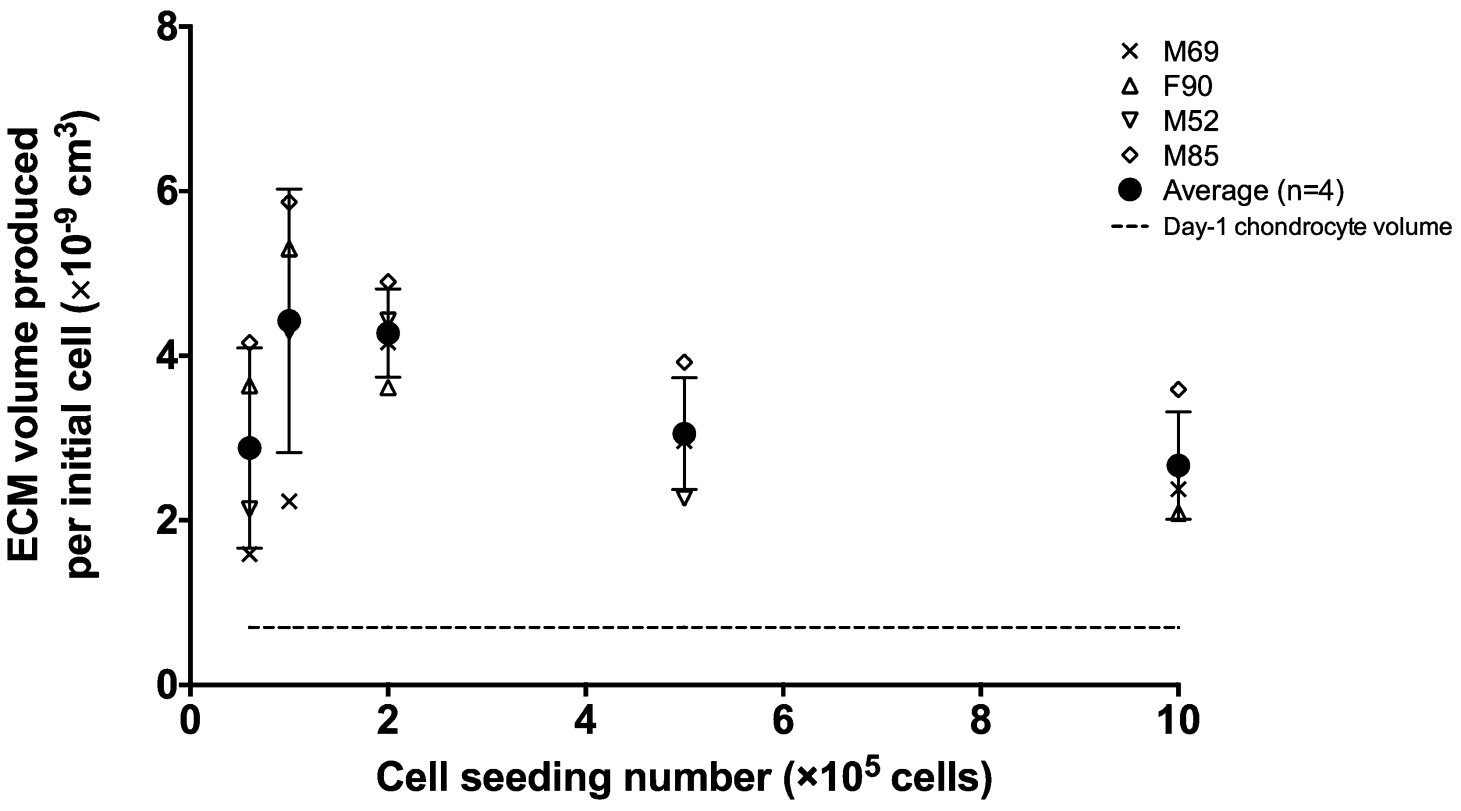
ECM synthesis per initial cell. The volume of ECM produced by cells contributed to the overall volume of Day-21 cartilage pellets. The maximum ECM volume increase per cell was observed at cell seeding numbers of 1 × 10^5^ and 2 × 10^5^.

Since no extracellular matrix (ECM) was present in the cell aggregate/pellet on Day 1, the volume increase in the cartilaginous pellets was primarily contributed by the volume of the ECM synthesized by the cells, in addition to any changes in cell number. Volume per initial cell was calculated as total volume of the Day-21 cartilaginous pellet divided by initial cell seeding number (comparison between initial cell seeding number and total cell number in Day-21 pellets is presented in supplementary Table S3). Consistent with the fold increase in cartilaginous pellet volume, the volume of ECM synthesized per cell was also observed to reach a maximum at initial cell seeding numbers of 1 × 10^5^ and 2 × 10^5^ (Fig. 2).

### Prediction of Oxygen Tension Profile Within Cartilaginous Pellet

**Figure 3:**
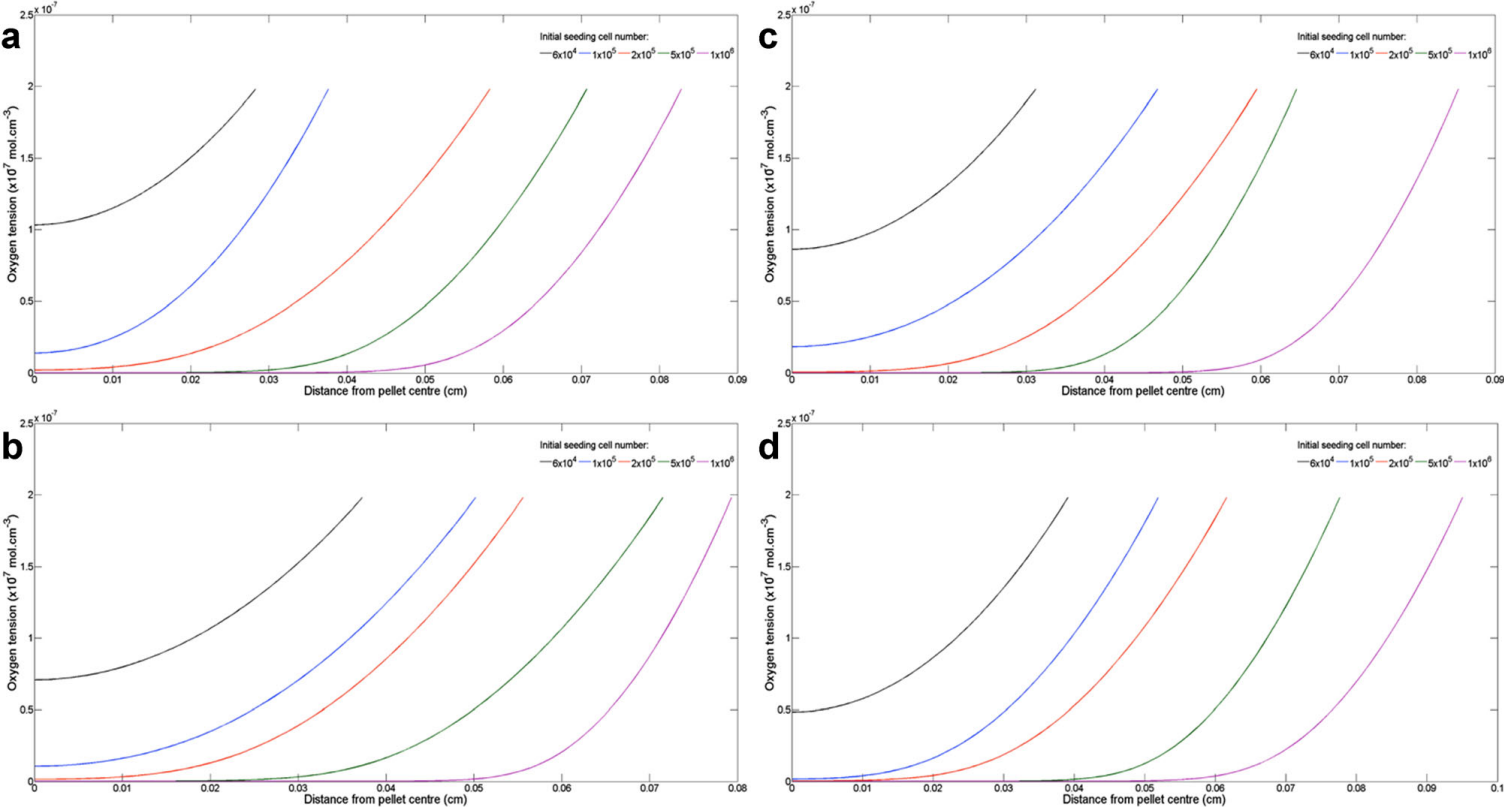
Prediction of oxygen tension profile within Day-21 cartilaginous pellets. The oxygen tension profiles were predicted for pellets generated using HACs from patient (a) M69, (b) F90, (c) M52, and (d) M85.

In the present study, key parameters in the mathematical model described earlier were determined experimentally. The BD OBS oxygen biosensor system was used to determine the patient-specific oxygen uptake rate (*Q*_cell_) of HACs (data shown in Table S2). Using the method described by Haselgrove et al. (1993), the Michaelis–Menten constant (*K*_m_), that is, the oxygen concentration at which half-maximum uptake rate (*Q*_cell_/2) occurred, was found to be 7.41 ± 0.32 × 10^−9^ [mol cm^−3^] based on the [O_2_] depletion curve over time. In addition, the cell density *ρ*_cell_, measured using a custom script in MatLab, was expressed as a function of distance from the pellet centre (*r*) (Fig. S6a–e). These parameters were substituted into the mathematical model for the prediction of local oxygen tension profile throughout the cartilaginous pellet (Fig. 3a–d). The predicted oxygen tension profile within Day-21 cartilaginous pellets was characterized by a gradual depletion in oxygen tension from the periphery towards the centre. Oxygen tension in the central region of Day-21 cartilaginous pellets generated using 6 × 10^4^ and 1 × 10^5^ cells did not reach zero. Anoxia (zero oxygen tension), however, was observed in pellets generated using cell seeding numbers of more than 2 × 10^5^. In particular, Day-21 cartilaginous pellets generated using 1 × 10^6^ cells were characterized by a rapid depletion of oxygen tension to anoxia.

### Evaluation of the Effect of O_2_ Tension on Cartilaginous Matrix Formation

**Figure 4:**
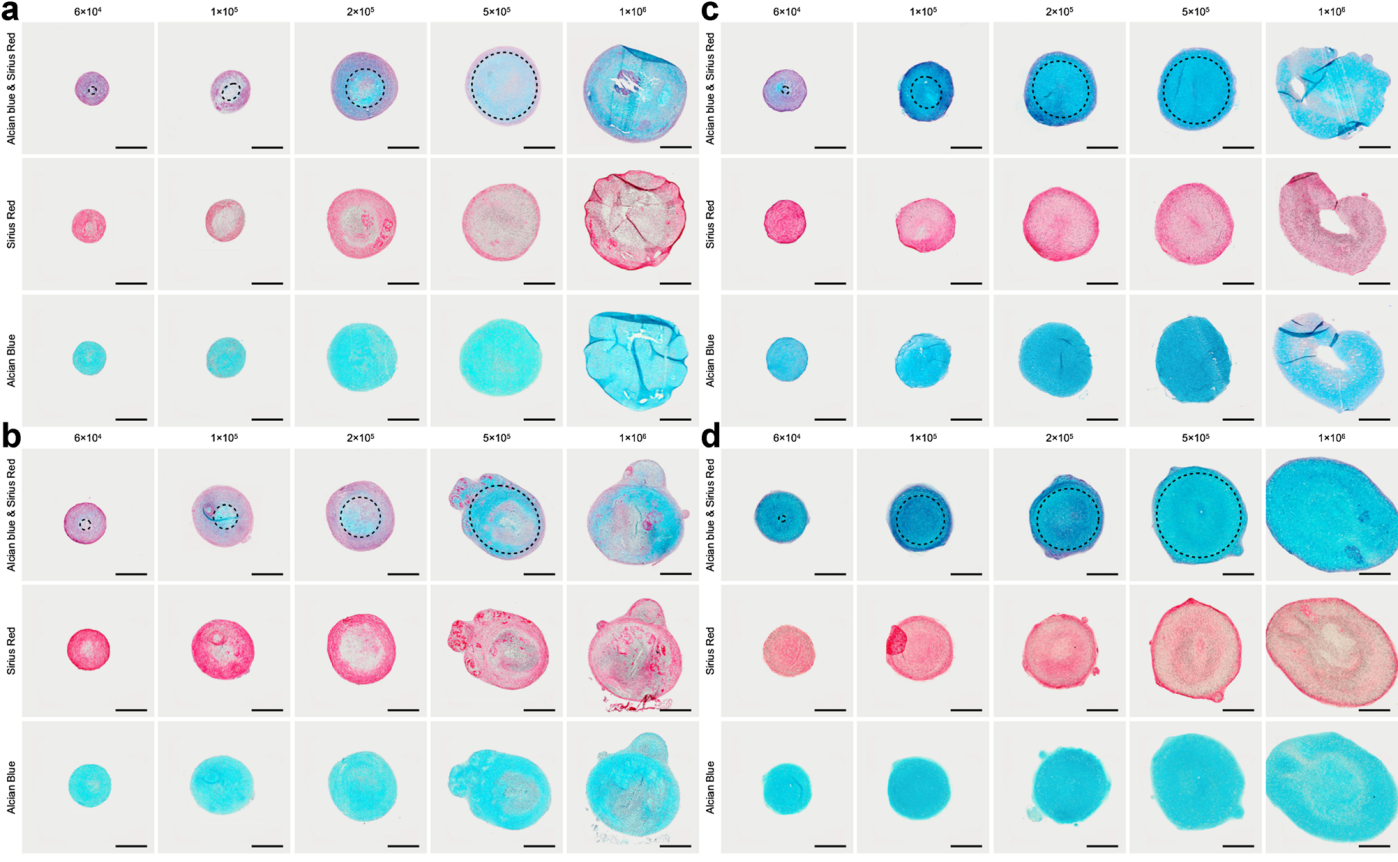
Cartilaginous pellet morphology demonstrated by Alcian blue and Sirius red staining. (a) M69, (b) F90, (c) M52, and (d) M85. In small-sized cartilaginous pellets generated using low cell seeding numbers (6 × 10^4^), strong Sirius red staining (collagen-rich ECM) was observed throughout the entire pellet. As the pellet size increased with initial cell seeding number (1 × 10^5^–5 × 10^5^), the amount of Alcian blue staining (PG content) also increased in proportion to collagen. This was visually demonstrated by the development of a thin distinct collagen-rich fibrous band (indicated by dotted lines) around the periphery surrounding the PG rich matrix. Excessively high cell seeding number (1 × 10^6^) resulted in the deformation and/or the development of a necrotic core, which was characterized by the lack of chondrogenic differentiation and matrix synthesis. Scale bars represent 200 µm.

**Figure 5:**
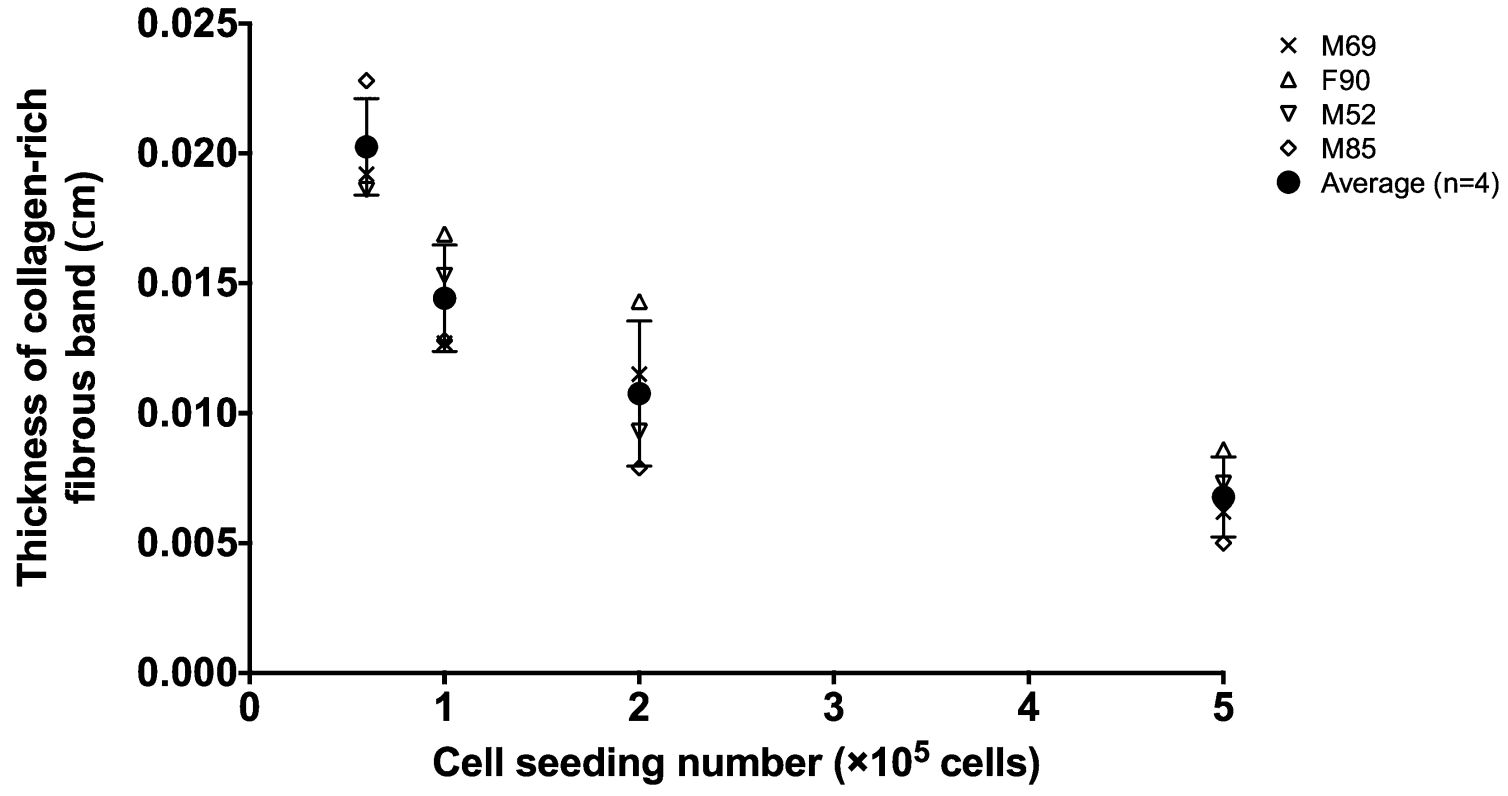
The thickness of the collagen-rich fibrous band around the pellet periphery was determined by ImageJ image analysis. Increasing initial cell seeding number (which resulted in increased pellet size) led to decreased fibrous collagenous band thickness. Pellets generated using 1 × 10^6^ cell seeding number were not included in this analysis due to the non-uniformity resulting from the irregular pellet geometry.

To elucidate the effect of oxygen tension on cartilaginous matrix formation in the pellet, the composition of the ECM was analyzed by Alcian blue and Sirius red staining (Fig. 4). Development of a distinct fibrous collagenous band (indicated by dotted lines in Fig. 4) was observed along the periphery of the pellet surrounding the proteoglycan (PG)-rich matrix. The thickness of the fibrous collagenous band was determined using ImageJ and presented in Figure 5. The increase in pellet size (as a result of increased initial cell seeding number) was accompanied by a reduction in the thickness of the fibrous collagenous band.

**Figure 6:**
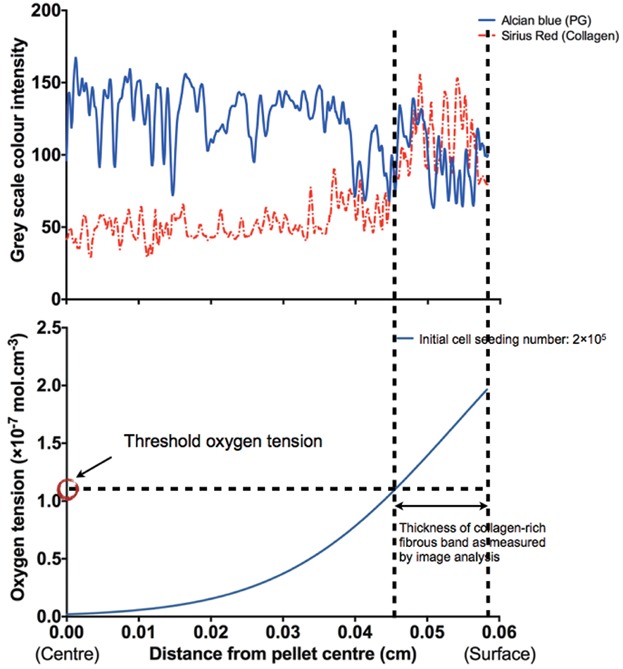
An example image demonstrating the methodology of determining the threshold oxygen tension. The thickness of fibrous collagenous band measured by ImageJ image analysis, color intensity profile and the O_2_ tension profile of Day-21 cartilaginous pellets were used for the extrapolation of the threshold oxygen tension. In this example (patient M69, initial cell seeding number = 2 × 10^5^), the average measured radius of pellets was 0.0583 cm. The average fibrous band thickness measured by ImageJ was 0.0115 cm. Therefore, the inner edge of collagen-rich fibrous band, that is, boundary between PG- and collagen-rich matrix, intersected at a distance of 0.0583 − 0.0115 = 0.0468 cm from the centre of the pellet. The threshold oxygen tension was then determined by reading the value of oxygen tension (1.10 × 10^−7^ mol cm^−3^) from the predicted oxygen tension profile at 0.0468 cm in this particular example. Three measurements were performed for each cell seeding number of each patient.

**Figure 7:**
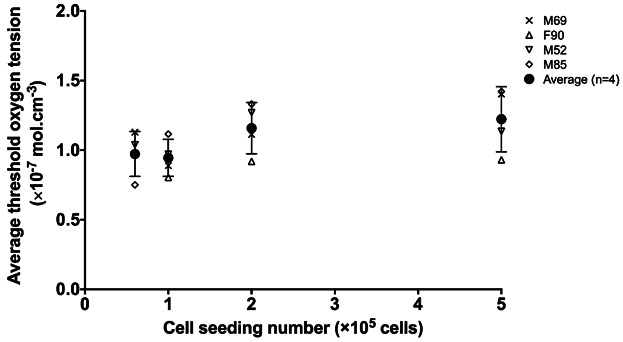
Threshold oxygen tension of human articular chondrocytes in “scaffold-free” pellet culture system. The average threshold oxygen tension was found to be consistent across all four patients and independent of the initial cell seeding number when patient-specific cellular oxygen uptake rate and pellet size were taken into account using the model. Pellets generated using 1 × 10^6^ cells were not included in the analysis due to their irregular shapes.

Similarly, the color intensity profiles of the Alcian blue and Sirius red-stained images clearly demonstrated the formation of a fibrous collagenous band along the periphery of the pellet, evidenced by extensive collagen (intense red stain) production in comparison to PG (blue stain) production (Fig. S7). By comparing the oxygen tension profiles to the histological analyses of cartilaginous pellets, it is possible to estimate a threshold oxygen tension (Fig. 6). We propose that the threshold oxygen tension can be used as a reference oxygen level, above which collagenous matrix production is favored and below which PG deposition is favored. Although there appeared to be a trend of increasing threshold oxygen tension with increasing initial cell seeding number (i.e., larger pellet size), the differences were not statistically significant (Fig. 7). In addition, the inter-patient variation observed was not statistically significant. Since pellets generated using 1 × 10^6^ cells were irregular in shape, they were excluded from calculation of the threshold oxygen tension and image analysis. The thickness of the fibrous collagenous band predicted using the average threshold oxygen tension of 8% was comparable to that determined experimentally (Fig. S8).

## Discussion

A combined experimental-computational approach, encompassing the 3D “scaffold-free” pellet culture technique, mathematical modeling and image analysis of stained histological sections, was adopted to determine the effect of oxygen tension on cartilaginous matrix synthesis by HACs. In osteoarthritis, degenerative changes in the articular cartilage are characterized by significant up-regulation of catabolic enzymes such as matrix metalloproteinases in chondrocytes of the superficial zone (Roach et al., 2005). Superficial zone chondrocytes were therefore excluded from the study. Only chondrocytes isolated from the deep zone of macroscopically healthy cartilage from the non load-bearing region of the femoral heads were utilized in the present study. In comparison to the scaffold-free approach, strategies using biomaterial scaffolds are highly relevant from a functional/mechanical point of view since scaffolds confer mechanical support upon implantation. The present study however has favored a “scaffold-free” approach over the use of biomaterial scaffolds, which may impair tissue formation due to unpredictable degradation rates and potential adverse immunogenicity of the degradation products therein (van der Kraan et al., 2002). More importantly, poorly designed biomaterials (e.g., lack of interconnected pores) can be a substantial barrier to nutrient penetration. It has been reported that the oxygen diffusion coefficient in polymers can be approximately 25 times lower in comparison to native cartilage tissue (Malda et al., 2004). Since a “scaffold-free” culture model was used in the work presented here, the cartilaginous pellet could be modeled as a single phase, thus eliminating the necessity of considering a separate biomaterial phase for the prediction of oxygen tension distribution within 3D cartilaginous pellets generated using 2D monolayer expanded HACs.

The mathematical model used in the present study was based on key parameters that were determined experimentally. Cellular oxygen uptake rate (*Q*_cell_) was an important parameter in the numerical prediction of the oxygen gradients within cartilaginous pellets. The method, that is, oxygen-sensitive fluorophore-based BD oxygen biosensor system, applied in the present study has been utilized in a series of studies to investigate the metabolism of chondrocytes/MSCs (Heywood and Lee, 2008, 2010; Heywood et al., 2006b, 2010; Pattappa et al., 2011). In the literature, as far as we are aware, no values have been reported for the oxygen uptake rate of primary HACs using the BD oxygen biosensor system. The values of *Q*_cell_ (average *Q*_cell_ = 4.20 ± 1.21 × 10^−17^ mol cell^−1^ s^−1^) for HACs determined in this study are of the same order (1 × 10^−17^) as previously reported, albeit Clarke electrodes were used (Johnson et al., 2000). The variation in the measured values of *Q*_cell_ may be due to a number of factors including inter-patient variation, extent of in vitro culture, culture conditions and gas exchange between sample and air through the plastic plate material (Arain et al., 2005; Heywood and Lee, 2008; Heywood et al., 2006b; Nikolaev et al., 2010; Pattappa et al., 2011).

Pellet radius (*r*_max_) and cell density (*ρ*_cell_) were also determined. In particular, instead of assuming a homogeneous cell distribution, depth-dependent cell distribution within the cartilaginous pellet was determined in the present study. Equations were fitted to describe *ρ*_cell_ in relation to the position (*r*) within the pellet, allowing accurate and tailored prediction of oxygen tension profiles for cartilaginous pellets with various sizes for each individual patient. In the “scaffold-free” pellet culture, initial cell seeding number directly affected the size of Day-21 cartilaginous pellets, that is, higher initial cell seeding number resulted in larger pellet size. Interestingly, the largest percentage volume increase (≈6-fold) was observed when initial cell seeding numbers of 1 × 10^5^ and 2 × 10^5^ were used. Similarly, the largest increase in ECM volume synthesized per initial cell was also observed when initial cell seeding numbers of 1 × 10^5^ and 2 × 10^5^ were used. The diffusion distance inevitably increased with the size of the cartilaginous pellets, preventing oxygen reaching the central region. The oxygen tension at the central region of pellets generated using 5 × 10^5^ and 1 × 10^6^ cells reached anoxic levels. This could be a limiting factor to the volume increase in larger cartilaginous pellets. Although chondrocytes are known to be well adapted to the hypoxic environment within native cartilage tissue in vivo, the importance of oxygen cannot be omitted completely in their principal function, namely, synthesis of cartilaginous ECM.

Despite the fact that articular chondrocytes can survive anoxic conditions (pO_2_ < 0.1% atmospheric pressure), adverse effects include severely compromised metabolic activity and matrix production (Grimshaw and Mason, 2000). Similar observations were made in the work presented here. A homogeneous cell distribution was observed in all pellets regardless of their sizes, however, histological analysis revealed distinct necrotic cores in central region of larger pellets resulting from cell seeding numbers above 5 × 10^5^ cells. The necrotic region was characterized by the lack of ECM deposition. It is therefore highly likely that although larger pellets can be generated using a high cell seeding number, optimal ECM synthesis by chondrocytes could only be achieved in smaller pellets where a certain level of oxygen was maintained.

It has been reported that both aerobic and anaerobic metabolic pathways are evident in cartilage constructs (Malda et al., 2003). Furthermore, it has been well documented that oxygen and glucose are closely interrelated in the so-called Crabtree effect and the negative Pasteur effect (Heywood et al., 2006a,b; Lee and Urban, 1997). Anoxic environments severely inhibit glycolysis and lactate production by articular chondrocytes. It is likely that impaired glycolysis, coupled with limited oxidative phosphorylation in anoxia, inevitably reduced ATP synthesis, resulting in the shortage of energy and subsequently the lack of cartilage ECM synthesis. Our results however indicate that high oxygen tension would favor fibrous collagenous tissue generation. High oxygen tension therefore may not necessarily provide optimal culture conditions for hyaline cartilaginous tissue formation.

In the current study, we observed that in regions at a certain distance from the pellet surface, the matrix predominantly consisted of fibrous collagen tissue, while in the inner region, the PG content was more prominent. The thickness of the visually distinguishable fibrous collagenous band was cross-referenced with the oxygen tension profile and, a threshold oxygen tension was determined. The threshold oxygen tension served as a reference boundary oxygen level, above which collagen production was favored, while a shift towards more PG production occurred when oxygen tension fell below this level. This was clearly demonstrated by histological analysis of cartilaginous pellet sections. In smaller pellets (e.g., 6 × 10^4^ cell seeding number), the regions experiencing oxygen tension higher than the threshold level covered a large distance from the pellet surface, resulting in more collagen tissue formation in the pellet. As the pellet size increased (e.g., 1–5 × 10^5^ cell seeding number), the regions experiencing high oxygen tension gradually decreased, resulting in the subsequent decrease in the thickness of collagen-rich fibrous band. Other studies have also suggested that the lack of oxygen can supress collagen synthesis in periosteal tissue explants as well as in tissue engineered cartilage (Obradovic et al., 1999; O'Driscoll et al., 1997). Sirius red staining, routinely applied for the visualization of fibrillar collagens including Type I to V, was used to identify the fibrous collagenous band in the present study. Cartilaginous pellets are characterized by the presence of Type I collagen in the outer rim of fibrous collagen, while the central hyaline cartilage-like region contains predominantly Type II collagen. Since histological sections of the cartilaginous pellets were not immunostained with antibodies against collagen I and II, the inability to localize and distinguish between the two collagen types is an acknowledged limitation of this study.

Despite the inter-patient variations in *Q*_cell_, pellet size, pellet morphology and oxygen tension profiles, which were key parameters involved in the determination of threshold oxygen tension, it is interesting to see that the values of threshold oxygen tension for all four patients demonstrated no statistical difference and were independent of cell seeding number. The average threshold oxygen tension measured in the present study was 1.075 (±0.364) × 10^−7^ mol cm^−3^ (pO_2_ ≈ 8% atmospheric pressure). To date, the reported effects of oxygen tension on chondrocyte proliferation and matrix synthesis in vitro remain highly controversial. While some studies have reported that chondrocyte matrix synthesis was enhanced by reduced oxygen tension (Jurgens et al., 2012; Katopodi et al., 2009; Saini and Wick, 2004), others have reported that hypoxic oxygen tension resulted in no/minimal differences in cell proliferation and/or chondrogenic differentiation in comparison to normoxia (Betre et al., 2006; Grimshaw and Mason, 2000). These reported differences in the response of articular chondrocytes to oxygen tensions appear largely due to species variation (e.g., bovine, human) and culture conditions (e.g., monolayer, bioreactor, or scaffolds), making sensible comparisons virtually impossible. Nevertheless, the findings in the present study, which hold true for HACs in “scaffold-free” pellet culture model, suggest that culture below 8% oxygen would reduce the formation of a collagenous outer layer and favor PG synthesis, as long as a sufficient level of oxygen in the centre of the pellet is maintained. It is however important to note that oxygen tension alone may not be the sole driving force for the formation of the fibrous collagenous band, and other contributing factors could include mechanical forces associated with cell spreading on the surface, fluid shear stress as well as soluble biochemical factors besides oxygen (Bueno et al., 2009; Kisiday et al., 2005).

The threshold oxygen tension was reported in this study with the caveat that its absolute accuracy was dependent on assumptions made for the mathematical model and measurements of key parameters such as *ρ*_cell_ and *Q*_cell_. The predicted oxygen tension profiles were dependent on the value of oxygen diffusion coefficient. Since the overall histology of HAC pellets was reminiscent of native hyaline cartilage, the oxygen diffusion coefficient was set at 1.5 × 10^−5^ cm^2^ s^−1^, that is, approximately 50% of the value in water, in light of values reported for intact native cartilage that range from 30% to 80% of the value in water (Haselgrove et al., 1993; Maroudas, 1979). By varying the oxygen diffusion coefficient to either 30% or 80% of the value in water, an approximately 25% change in the measured threshold oxygen tension was observed (data not shown). Constructs maintained in tubes in static culture conditions have been demonstrated to be subjected to longer diffusion pathways, which could potentially reduce oxygen availability (Sengers et al., 2005b). Another important assumption was that the cell culture media was considered to be well mixed, that is, always in equilibrium with ambient oxygen tension. Lower ambient oxygen tension in the culture media would result in lower estimation of threshold oxygen tension. In vivo, cyclic mechanical loading of cartilage can influence the rate at which large solutes such as growth factors, hormones, and cytokines are transported to cells (O'Hara et al., 1990). This however is only relevant for large molecules, since mechanically induced fluid velocities are too low to significantly enhance the transport of small molecules such as oxygen and glucose over diffusion alone (Sengers et al., 2004).

Sensitivities of the model-predicted oxygen tension profile to changes in the parameters *ρ*_cell_ and *Q*_cell_ were analyzed. Due to the relatively slow proliferation rate of chondrocytes (Lee et al., 1998), the initial cell seeding number could have been used directly to provide an estimate for the homogeneous *ρ*_cell_ in Day-21 cartilaginous pellets. The discrepancy between predicted oxygen tension profiles using a homogenous *ρ*_cell_ and the actual experimentally determined *ρ*_cell_ was less than 5% for all pellet sizes and patient samples (data not shown). Therefore, the model is less sensitive to changes in the *ρ*_cell_. The maximum *Q*_cell_ was increased or decreased by 50% of its original fitted value. The impact of changing the *Q*_cell_ term led to a greater than 30% change in the measured threshold oxygen tension (data not shown). This significant impact highlighted the importance of accurate measurement of patient specific *Q*_cell_.

In the model, oxygen consumption was assumed to follow Michaelis–Menten kinetics to represent the dependence of *Q*_cell_ upon oxygen concentration alone (Galban and Locke, 1999; Haselgrove et al., 1993; Zhou et al., 2004). However, oxygen uptake is also affected by low glucose and pH, and this may alter the prediction of oxygen tension profiles, depending on culture conditions, that is, static, perfusion, and suspended cultures (Zhou et al., 2008). Moreover, dedifferentiation of HACs during 2D monolayer expansion can alter *Q*_cell_. Chondrocytes in the native 3D in vivo environment maintain a glycolytic metabolism even under aerobic conditions (known as the Warburg effect), while proliferative chondrocytes in monolayer cultures under aerobic conditions gradually develop an oxidative metabolism with increased oxygen uptake (Heywood and Lee, 2008; Heywood et al., 2010).

The present study only investigated the potential relationship between oxygen tension and matrix synthesis by HACs in a pellet culture system. In addition to oxygen, glucose concentration has been shown to affect glycosaminoglycan (GAG) synthesis by chondrocytes in tissue engineered cartilage (Heywood et al., 2006a). Since nutrient supply and metabolic waste removal rely on diffusion in tissue engineered constructs (especially, avascular cartilage), gradients of nutrients, and waste metabolites exist through the constructs. Therefore, it has to be emphasized that gradients of other nutrients (glucose) and waste metabolites (lactate) could affect matrix production in the tissue engineered constructs. Finally, the model used in the present study does not provide insight into how the temporal evolution of the spatial distribution of oxygen governs the growth process of cartilaginous pellets from Day 1 to Day 21 (Lewis et al., 2005), which will need to be investigated in future studies.

## Conclusion

The effects of oxygen tension on cartilaginous ECM synthesis by HACs were examined in the present study. It was observed that the syntheses of the two major cartilaginous ECM constituents, namely collagen and PG, required distinct oxygen tensions. The mathematical model served as a predictive tool, from which a threshold oxygen tension was found. Oxygen tensions above the threshold level were observed to favor collagenous matrix production, whereas oxygen tensions below this level enhanced PG deposition. The combined experimental-computation approach is important for improved understanding of chondrogenesis and optimization of culture environments for cartilage tissue engineering.

The authors gratefully acknowledge financial support for the work from the Faculty of Medicine Research Management Committee to R.S.T., studentship support to S.L. from the University of Southampton PhD scholarship, EPSRC (EP/H028277/1) funding for B.G.S., BBSRC (G0105791/1) funding for R.O.C.O. The authors would like to thank Dr. Fay Chinnery and Dr. Franchesca Houghton for their assistance with the BD OBS oxygen biosensor system and Orthopaedic surgeons at Southampton General Hospital for provision of femoral head samples.
