# Supplementary material for: The effect of oxygen tension on human articular chondrocyte matrix synthesis: Integration of experimental and computational approaches
Source: Biotechnol Bioeng. 2014 May 5;111(9):1876–85. doi: 10.1002/bit.25241 (PMC4284020; doi:10.1002/bit.25241)

Table S - 1. The radii of day-21 HAC cartilaginous pellets.

Table S - 2. Cellular oxygen uptake rate (Q_cell_) of HACs measured using BD Oxygen Bioensor System.

Table S - 3. Comparison between initial cell seeding number and total cell number in day-21 cartilaginous pellets.

Figure S - 1. Determination of surface area (SA_sec_) and radius (r_sec_) of a cartilaginous pellet section. The “Analyse Particle” tool in ImageJ was used to trace the entire outline and determine the enclosed surface area of a cartilaginous pellet section (SA_sec_). The radius of an individual pellet section was calculated from the circle with the same SA_sec_, i.e. $\mathbf{r}_{\mathbf{sec}}\mathbf{=}\sqrt{\mathbf{SA}_{\mathbf{sec}}\boldsymbol{/\pi}}$. In this example, the SA_sec_ of the pellet section was denoted as A1 (12.51×10^5^μm^2^), and therefore the r_sec_ was calculated as: $\mathbf{r}_{\mathbf{sec}}\mathbf{=}\sqrt{\mathbf{SA}_{\mathbf{sec}}\boldsymbol{/\pi}}\mathbf{=}\sqrt{\boldsymbol{12.51\times}\mathbf{10}^{\mathbf{5}}\mathbf{/3.14}}\boldsymbol{=631.04 \mu m}$. Scale bar represent 200μm.

Figure S - 2. Identification of cell nuclei using MatLab. Sections stained with only Sirius red were used due to high contrast between cell nuclei and background (a). A custom programmed script in MatLab was used to identify cell nuclei counter-stained with Weigert’s haematoxylin (b). Local cell density was determined in zonal regions with equal width of 50μm (c). Scale bar represent 200μm.

Figure S - 3. Image reconstruction using MatLab. The colours of interest, Alcian blue (PG) and Sirius red (collagen), were recreated using grey-scale pixels. Top: Sirius red staining. Bottom: Alcian blue staining. (a) Original colour images of stained pellet sections. (b) Single channel images. (c) Reconstructed grey-scale images.

Figure S - 4. Measurement of fibrous collagenous band thickness around pellet periphery. Reconstructed Sirius red images were used for the measurement using ImageJ. Measurement selection tools (yellow lines) were placed at 0°, 45°, 90°, 135°, 180°, 225°, 270° and 315° positions around the pellet periphery. The average thickness of the collagenous band was then calculated from these 8 measurements.

Figure S - 5. Graph of oxygen tension versus time during the 120-minute measurement period used to determine the cellular oxygen uptake rate. The graph shown here is for chondrocytes (500,000 cells) from patient M69.

Figure S - 6. Cell density distribution within cartilaginous pellets. Initial cell seeding number: (a) 6×10^4^, (b) 1×10^5^, (c) 2×10^5^, (d) 5×10^5^ and (e) 1×10^6^. For each patient and initial cell seeding number, cell density (ρ_cell_ ) was expressed as a function of distance from pellet centre (r) for the model prediction of oxygen tension profile.

Figure S - 7. Intensity profile of Sirius red and Alcian blue stained images of cartilaginous pellet sections determined using ImageJ. Initial cell seeding number: (a-d) 6×10^4^, (e-h) 1×10^5^, (i-l) 2×10^5^ and (m-p) 5×10^5^. Patient details: (a, e, i, m) M69, (b, f, j, n) F90, (c, g, k, o) M52 and (d, h, l, p) M85.

Figure S - 8. The thickness of the fibrous collagenous band predicted using the average threshold oxygen tension of 8% was comparable to that determined experimentally. Model predictions were based on oxygen profiles calculated for each patient (a – M69, b – F90, c – M52, d – M85) using the measured patient-specific oxygen uptake rate and pellet size.

Table S1

| **Patient details** | **Initial cell seeding number** | **Day-21 cartilaginous pellet radius [cm]** | **Patient details** | **Initial cell seeding number** | **Day-21 cartilaginous pellet radius [cm]** |
| --- | --- | --- | --- | --- | --- |
| M69 | 6×10^4^ | 0.0283 | M52 | 6×10^4^ | 0.0312 |
|  | 1×10^5^ | 0.0376 |  | 1×10^5^ | 0.0468 |
|  | 2×10^5^ | 0.0583 |  | 2×10^5^ | 0.0595 |
|  | 5×10^5^ | 0.0707 |  | 5×10^5^ | 0.0646 |
|  | 1×10^6^ | 0.0828 |  | 1×10^6^ | 0.0853 |
| F90 | 6×10^4^ | 0.0373 | M85 | 6×10^4^ | 0.0391 |
|  | 1×10^5^ | 0.0502 |  | 1×10^5^ | 0.0519 |
|  | 2×10^5^ | 0.0556 |  | 2×10^5^ | 0.0616 |
|  | 5×10^5^ | 0.0715 |  | 5×10^5^ | 0.0776 |
|  | 1×10^6^ | 0.0793 |  | 1×10^6^ | 0.0950 |

Table S2

| **Patient details** | **Maximum cellular O2 uptake rate [mol.cell^-1^.s^-1^]** |
| --- | --- |
| M69 | 3.02±0.27 × 10^-17^ |
| F90 | 5.88±0.43 × 10^-17^ |
| M52 | 3.76±0.28 × 10^-17^ |
| M85 | 4.12±0.31 × 10^-17^ |

Table S3

| **Patient details** | **Cell seeding number** | **Total cell number in day-21 pellets** |
| --- | --- | --- |
| M69 | 6×10^4^ | 54915 |
|  | 1×10^5^ | 135347 |
|  | 2×10^5^ | 261625 |
|  | 5×10^5^ | 438560 |
|  | 1×10^6^ | 885505 |
| F90 | 6×10^4^ | 87440 |
|  | 1×10^5^ | 130887 |
|  | 2×10^5^ | 241127 |
|  | 5×10^5^ | 376176 |
|  | 1×10^6^ | 706226 |
| M52 | 6×10^4^ | 66436 |
|  | 1×10^5^ | 130887 |
|  | 2×10^5^ | 241127 |
|  | 5×10^5^ | 467982 |
|  | 1×10^6^ | 950643 |
| M85 | 6×10^4^ | 99655 |
|  | 1×10^5^ | 176544 |
|  | 2×10^5^ | 253153 |
|  | 5×10^5^ | 445365 |
|  | 1×10^6^ | 941544 |

Figure S-1


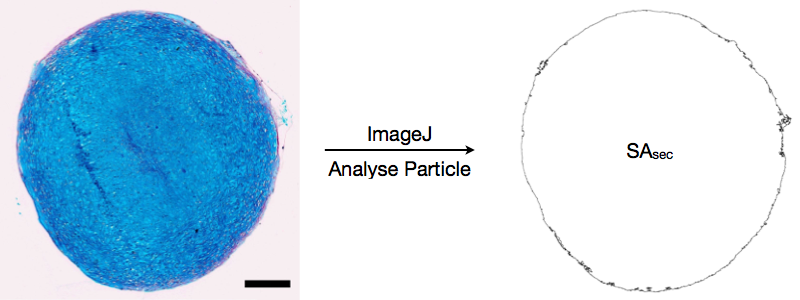


Figure S-2


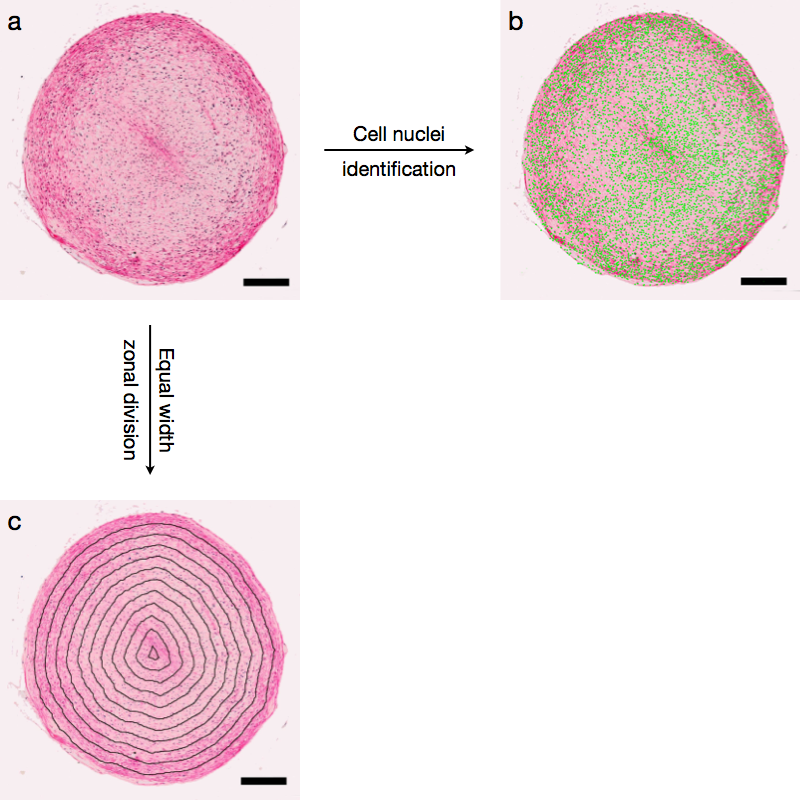


Figure S-3


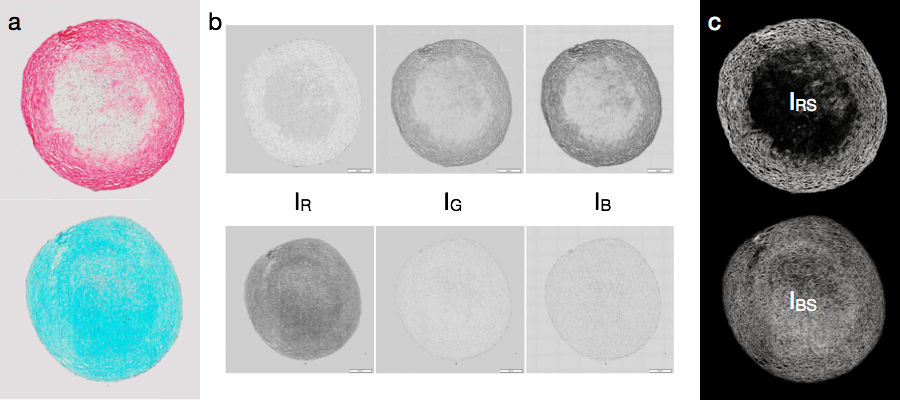


Figure S-4


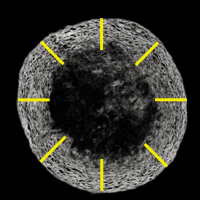


Figure S-5


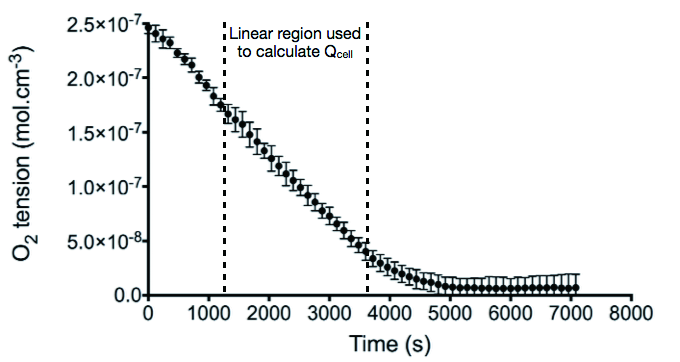


Figure S-6


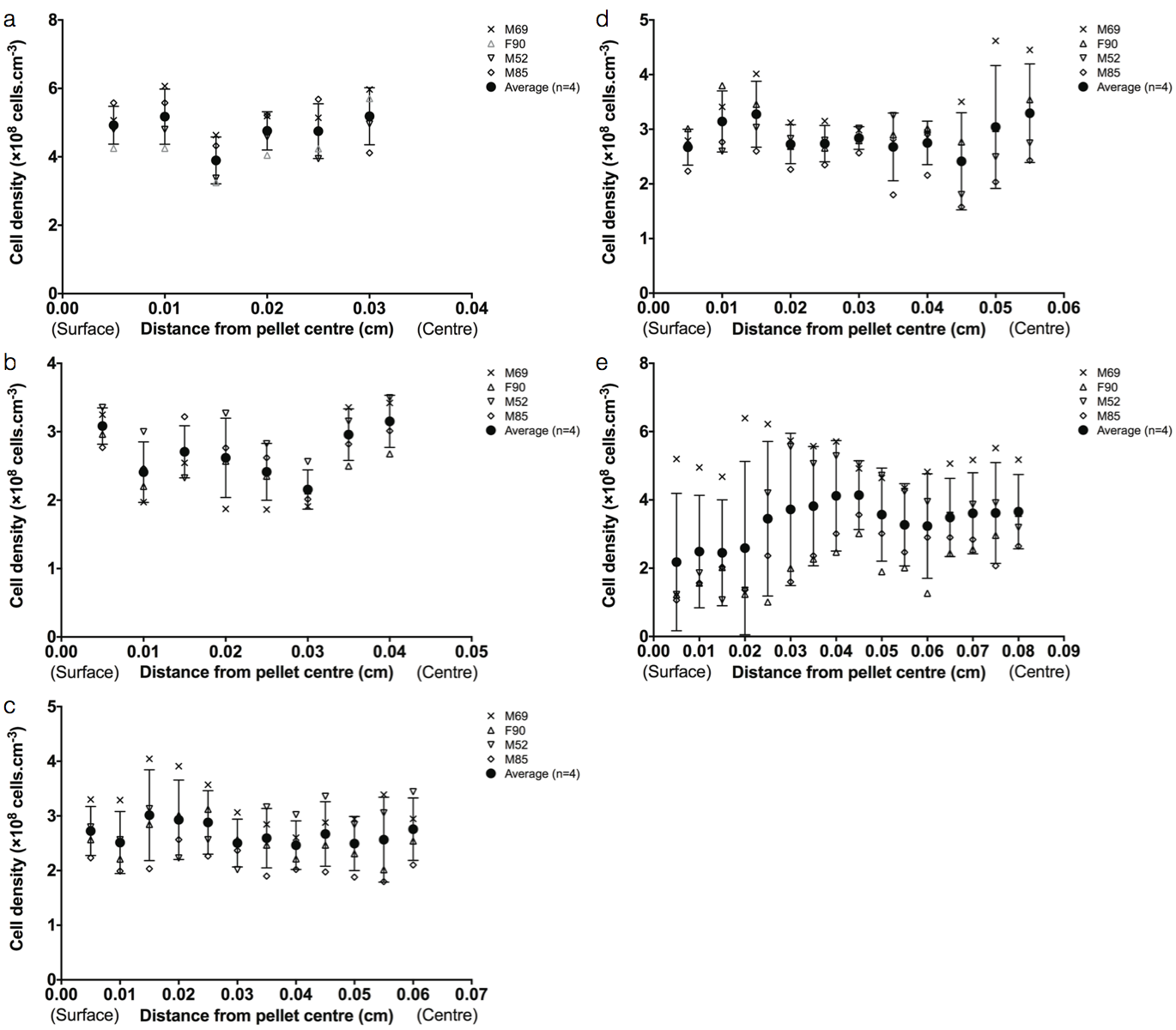


Figure S-7


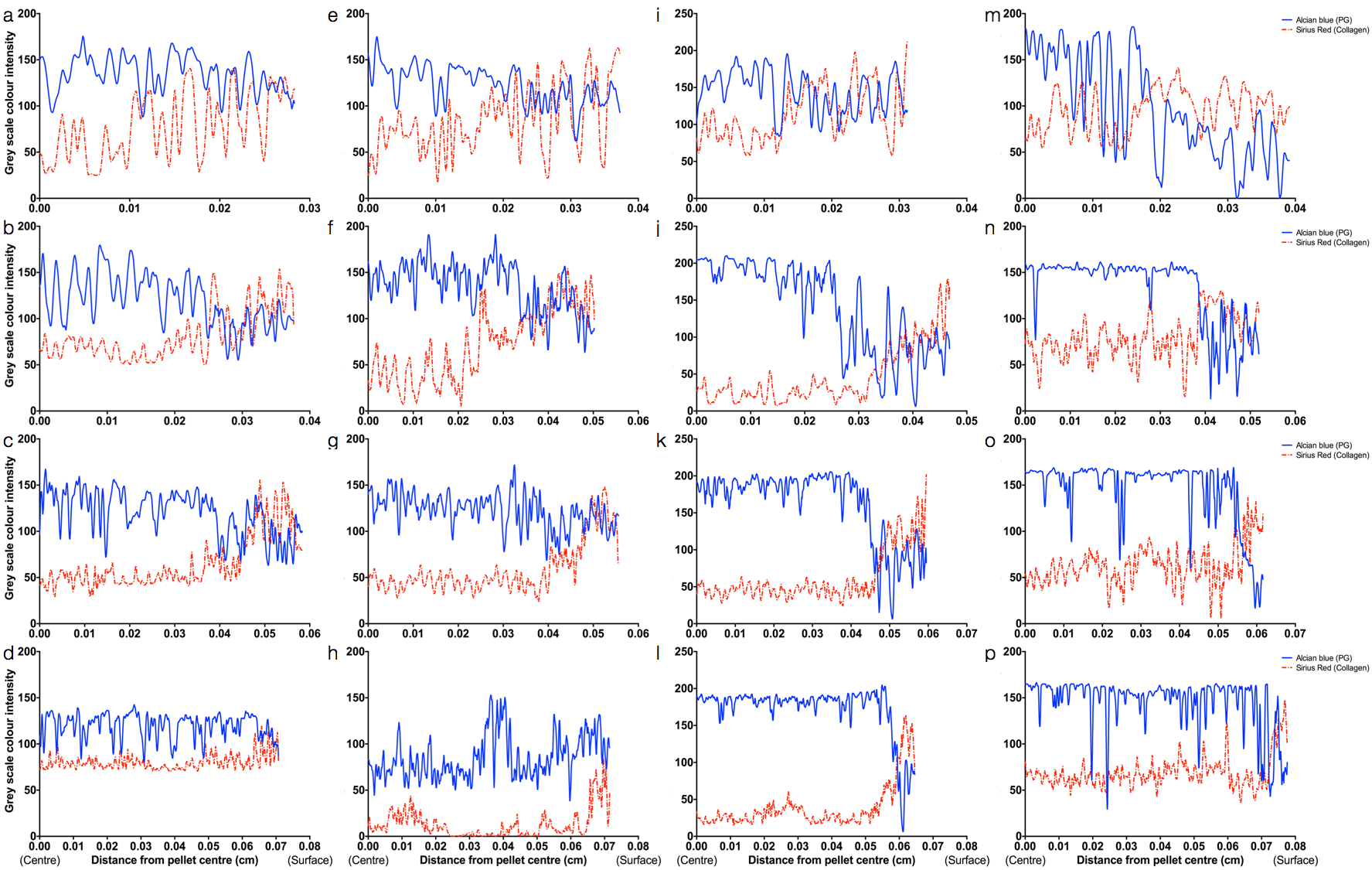


Figure S-8


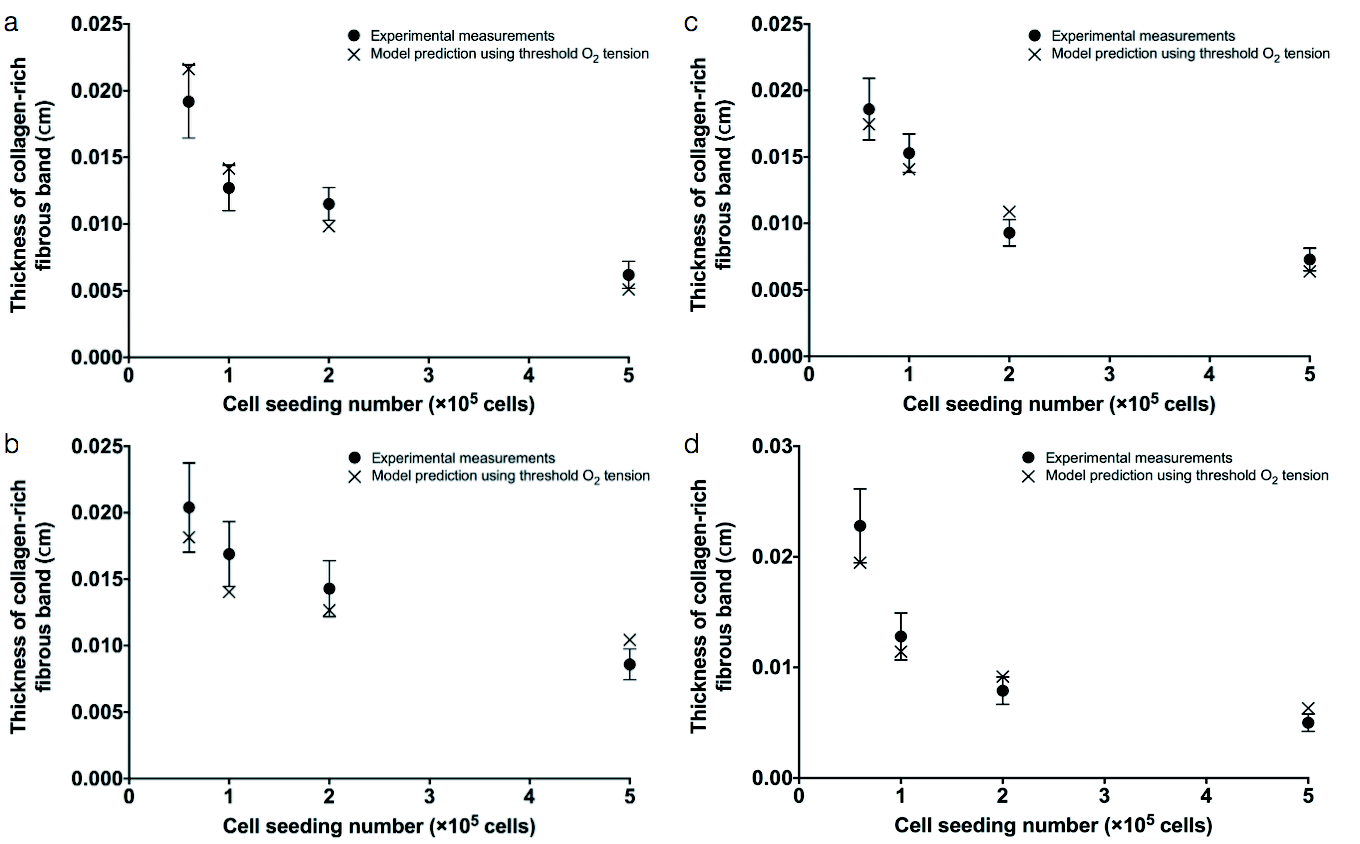

Supplement: Supplementary file 1 [file bit0111-1876-SD1.docx]
